# Two Seemingly Homologous Noncoding RNAs Act Hierarchically to Activate *glmS* mRNA Translation

**DOI:** 10.1371/journal.pbio.0060064

**Published:** 2008-03-18

**Authors:** Johannes H Urban, Jörg Vogel

**Affiliations:** Max Planck Institute for Infection Biology, RNA Biology Group, Berlin, Germany; Howard Hughes Medical Institute Janelia Farm, United States of America

## Abstract

Small noncoding RNAs (sRNA) can function as posttranscriptional activators of gene expression to regulate stress responses and metabolism. We here describe the mechanisms by which two sRNAs, GlmY and GlmZ, activate the *Escherichia coli glmS* mRNA, coding for an essential enzyme in amino-sugar metabolism. The two sRNAs, although being highly similar in sequence and structure, act in a hierarchical manner. GlmZ, together with the RNA chaperone, Hfq, directly activates *glmS* mRNA translation by an anti-antisense mechanism. In contrast, GlmY acts upstream of GlmZ and positively regulates *glmS* by antagonizing GlmZ RNA inactivation. We also report the first example, to our knowledge, of mRNA expression being controlled by the poly(A) status of a chromosomally encoded sRNA. We show that in wild-type cells, GlmY RNA is unstable due to 3′ end polyadenylation; whereas in an *E. coli pcnB* mutant defective in RNA polyadenylation, GlmY is stabilized and accumulates, which in turn stabilizes GlmZ and causes GlmS overproduction. Our study reveals hierarchical action of two well-conserved sRNAs in a complex regulatory cascade that controls the *glmS* mRNA. Similar cascades of noncoding RNA regulators may operate in other organisms.

## Author Summary

Hierarchical action of regulators is a fundamental principle in gene expression control, and is well understood in protein-based signaling pathways. We have discovered that small noncoding RNAs (sRNAs), a new class of gene expression regulators, can also act hierarchically and form a regulatory cascade. Two highly similar sRNAs function after transcription to activate the *Escherichia coli glmS* mRNA, which codes for an essential function in amino-sugar metabolism. It is somewhat unusual for two sRNAs to act upon the same target mRNA, and despite their seeming homology, these two sRNAs (GlmY and GlmZ) employ different molecular mechanisms and function hierarchically to activate *glmS* expression: GlmZ directly activates *glmS* translation via disruption of an mRNA structure that inhibits translation, whereas GlmY controls the processing of GlmZ to prevent the inactivation of this direct activator. We also found that GlmY is itself controlled by an RNA processing event (3′ end polyadenylation), which typically destabilizes bacterial RNA. Our data unequivocally demonstrate that *E. coli glmS* is exceptionally dependent on RNA-based mechanisms for its genetic control. Given the large number of noncoding RNAs of unknown function, we believe that similar regulatory RNA cascades may operate in other organisms.

## Abstract 3

A regulatory RNA cascade that posttranscriptionally activates the *glmS* mRNA is identified, with two highly similar small noncoding RNAs acting hierarchically in a manner thus far known only in protein-based regulatory circuits.

## Introduction

The posttranscriptional regulation of *glmS* expression, a gene encoding an essential enzyme (glucosamine-6-phosphate [GlcN-6-P] synthase) in amino-sugar metabolism, has recently attracted much attention. In Bacillus subtilis, the 5′ untranslated region (5′ UTR) of *glmS* mRNA contains a ribozyme that undergoes self-cleavage in the presence of GlcN-6-P, thereby destabilizing the *glmS* mRNA [1]. This *cis*-encoded mechanism of *glmS* mRNA control and metabolite sensing seems to be highly conserved among Gram-positive species [2]. Whereas Gram-negative bacteria such as E. coli lack the *glmS* ribozyme [2], recent studies have shown that *E. coli glmS* is posttranscriptionally controlled by multiple *trans*-encoded factors, including small noncoding RNAs (sRNAs) and several proteins [3–5].

**Figure 1:**
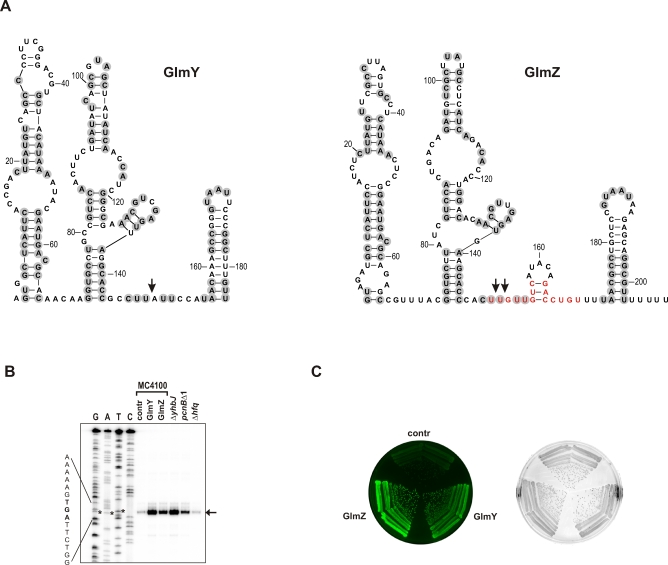
GlmY and GlmZ Are Related sRNAs and Promote GlmS Synthesis (A) Consensus structures of the E. coli GlmY (184 nt) and GlmZ (207 nt) RNAs (see [3] and Figure S1, respectively, for sequence alignments). Vertical arrows indicate previously mapped 3′ processing sites [6,9]. Grey circles denote nucleotides conserved between the E. coli GlmY and GlmZ RNAs. The GlmZ residues involved in *glmS* mRNA binding (see Figure 3A below) are shown in red. (B) Primer extension analysis detects the 5′ end of monocistronic *glmS* mRNA (vertical arrow) that results from processing of dicistronic *glmUS* mRNA at the *glmU* stop codon (UGA; denoted by asterisks). Total RNA was prepared from early stationary phase cultures of E. coli strain MC4100 carrying control vector pJV300 (lane “contr”), or the sRNA expression plasmids, pP_L_-GlmY (lane “GlmY”) or pP_L_-GlmZ (lane “GlmZ”), and from strains JVS-8018 (Δ*yhbJ*), JVS-2058 (*pcnB*Δ1), or Δ*hfq::cat*, as indicated. A 5′ end-labeled primer complementary to the *glmS* coding region was annealed to RNA, followed by reverse transcription and separation of the obtained cDNAs on a sequencing gel along with a corresponding sequencing ladder (lanes G, A, T, and C). Shown is an autoradiogram of the relevant section of the gel. (C) Both GlmY and GlmZ activate a translational *glmS::gfp* fusion. Colony fluorescence of E. coli carrying a *glmS::gfp* fusion plasmid (pJU-172) in combination with plasmid pJV300, pP_L_-GlmY, or pP_L_-GlmZ. Labeling as in (B). Shown are images of a LB agar plate obtained in the fluorescence mode (left) or the visible light mode (right).

Functional characterization of two E. coli sRNAs, GlmY and GlmZ (a.k.a. SroF/tke1 [6,7] and RyiA/SraJ [8,9], respectively; Figure 1A) identified these as positive regulators of *glmS* mRNA; constitutive expression of either sRNA causes overproduction of GlmS protein [3,4]. In addition, the bacterial Sm-like protein, Hfq, a facilitator of sRNA–mRNA interactions [10,11], has been implicated in GlmZ RNA function. Hfq associates with GlmZ RNA in vivo [8], and is required for the GlmZ-mediated up-regulation of GlmS protein synthesis [4].

**Figure 3:**
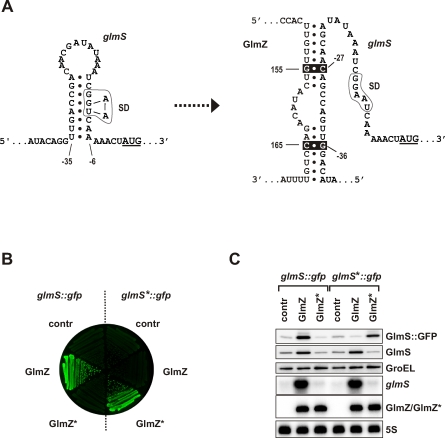
GlmZ Directly Acts on the *glmS* mRNA to Enhance GlmS Synthesis (A) Predicted anti-antisense mechanism by which GlmZ activates the *E. coli glmS* mRNA. The left panel shows the 5′ UTR of the *glmS* mRNA in an unbound, “inactive” state in which an intrinsic hairpin sequesters the *glmS* SD (boxed) and thus inhibits translation. Residues −42 to +3 relative to the AUG start codon, which is underlined, are shown. The right panel shows *glmS* mRNA in its “activated” form, i.e., upon base-pairing of residues 150–157 and 163–169 of GlmZ RNA with the *glmS* 5′ UTR, which disrupts the inhibitory hairpin and liberates the *glmS* SD. The positions of point mutations introduced in *glmS* (C_−27_ → G, G_−36_ → C; glmS* allele) and GlmZ (G_+155_ → C, C_+165_ → G; GlmZ* allele), which are expected to maintain base pairing in the glmS*/GlmZ* duplex, are indicated by inverse (white on black) print. (B) Colony fluorescence of strain JVS-8113 (Δ*glmY* Δ*glmZ*) carrying either the *glmS::gfp* or glmS*::gfp fusion plasmids, each in combination with plasmid pJV300 (“contr”), pP_L_-GlmZ (“GlmZ”), or pP_L_-GlmZ* (“GlmZ*”). Shown is an image of a LB agar plate obtained in the fluorescence mode. (C) The strains shown in (B) were grown to early stationary phase and subjected to western blot (top three panels) and northern blot (bottom three panels) analysis. The plasmid-encoded GlmS::GFP fusion protein was detected using α-GFP antibodies (top panel); the chromosomally encoded *glmS* mRNA and GlmS protein, and the plasmid-encoded GlmZ RNA (as indicated) were detected as in Figure 2.

**Figure 2:**
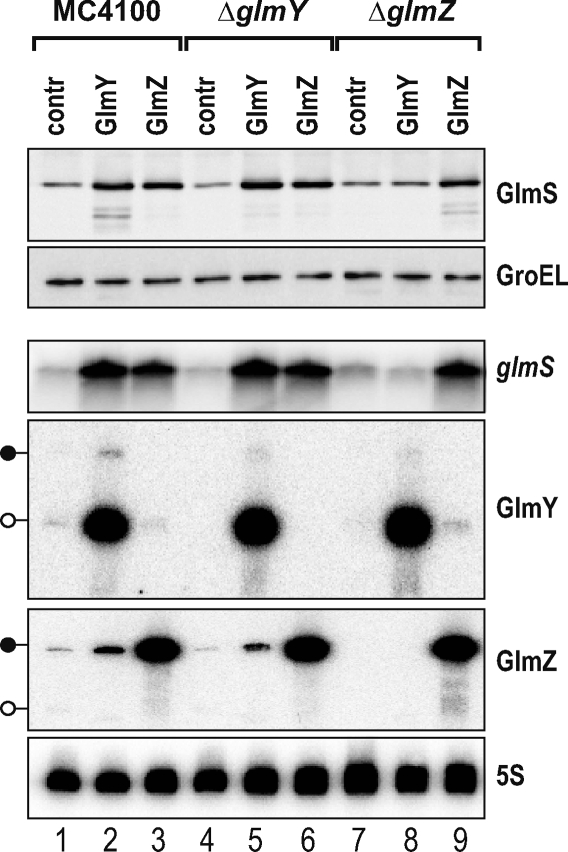
GlmY-Mediated *glmS* Activation Strictly Requires *glmZ* Western and northern blot analysis of strains MC4100, JVS-8030 (Δ*glmY*), or JVS-8024 (Δ*glmZ*), each of these strains carrying plasmids pJV300 (“contr” lanes), pP_L_-GlmY (“GlmY” lanes), or pP_L_-GlmZ (“GlmZ” lanes). Whole-cell protein and total RNA samples were prepared from early stationary phase cultures. Western blots were probed with sera specific for GlmS (top panel) or GroEL (loading control; second panel from top) proteins. For northern blot detection of *glmS* mRNA (third panel from top), 12 μg of total RNA per lane was separated on a 1.5% agarose-formaldehyde gel. To detect GlmY and GlmZ, 5 μg of total RNA was separated on a denaturing 5% PAA gel (fourth and fifth panels from top) as shown. The 5S rRNA (bottom panel) probing confirmed equal amounts of RNA in each sample. Black lollipops indicate the primary GlmY or GlmZ transcripts, whereas open lollipops indicate the processed, approximately 150-nt species of these sRNAs.

The PAP I and YhbJ proteins negatively regulate *glmS* mRNA expression; E. coli strains carrying functional disruptions of the respective genes overproduce *glmS* mRNA and GlmS protein [4,5]. PAP I (poly(A) polymerase I encoded by *pcnB*) is the main enzyme responsible for 3′ end RNA polyadenylation [12], which typically destabilizes bacterial transcripts by facilitating the 3′ → 5′ exonucleolytic degradation of RNA with a structured 3′ end [13,14]. Interestingly, *glmS* mRNA is markedly stabilized in a PAP I–deficient strain, i.e., *E. coli pcnB*Δ1 [5]. However, it has been unclear whether *glmS* mRNA is a substrate of PAP I, and whether polyadenylation *directly* destabilizes this mRNA in wild-type cells. YhbJ is a protein of unknown function, and appears to act upstream of GlmZ to negatively regulate *glmS* mRNA [4]. That is, the GlmS overproduction observed in an E. coli Δ*yhbJ* mutant strain is lost upon *glmZ* deletion [4].

Collectively, these observations suggested that multiple factors encoded by unlinked loci of the E. coli chromosome control *glmS* epistasis at the posttranscriptional level. However, whether these factors acted in concert or independently, and how GlmS protein synthesis was ultimately activated, remained to be determined. Here, we show that the regulatory GlmY and GlmZ sRNAs display significant homology of sequence and structure. Despite this apparent homology, we have found that the two RNAs act hierarchically such that GlmY acts upstream of GlmZ, and only GlmZ directly activates *glmS* mRNA translation. We propose that the main role of GlmY in this novel regulatory RNA cascade is to antagonize YhbJ-dependent inactivation of GlmZ RNA, i.e., the bona fide *glmS* activator. We also show that PAP I controls the levels of GlmY, and that loss of GlmY RNA polyadenylation causes the previously observed GlmS overproduction in PAP I–deficient E. coli. The hierarchical action of noncoding RNAs described here reveals a novel principle in RNA-based regulatory circuits previously only known in protein-based systems.

## Results

### Similarities of GlmY and GlmZ RNAs

*E. coli glmS* is transcribed as part of a dicistronic *glmUS* operon mRNA [15]. Ribonuclease E cleaves the *glmUS* transcript within the *glmU* stop codon; this event generates a monocistronic *glmS* mRNA which strongly accumulates in *E. coli pcnB*Δ1 or Δ*yhbJ*, and causes GlmS overproduction [4,5]. We used primer extension assays to test whether constitutive GlmY or GlmZ expression also induces the accumulation of monocistronic *glmS* mRNA. To this end, E. coli strain MC4100 (wild type) was transformed with plasmids pP_L_-GlmY or pP_L_-GlmZ carrying the *glmY* or *glmZ* genes, respectively, under control of a constitutive P_LlacO1_ promoter. Figure 1B shows that the expression of either sRNA promotes the accumulation of a monocistronic *glmS* mRNA species identical to that observed in Δ*yhbJ* or *pcnB*Δ1 mutant strains.

We previously established several *glmS::gfp* fusion plasmids as reporters of GlmY activity [3]. Of these, plasmid pJU-172 constitutively expresses a *glmS::gfp* fusion mRNA spanning the C-terminal *glmU* sequence (last 17 codons), the *glmS* 5′ UTR (161 nucleotides [nt]), and the first seven *glmS* codons translationally fused to *gfp*. This fusion is highly activated in the presence of the *glmY* expression plasmid, pP_L_-GlmY, suggesting that GlmY activates the *glmS* gene at the posttranscriptional level in a process that involves the *glmS* 5′ UTR. To test whether this fusion was also activated by GlmZ, the colony fluorescence of E. coli carrying pJU-172 in combination with a control plasmid (pJV300), or pP_L_-GlmY or pP_L_-GlmZ, was compared (Figure 1C). GlmZ activated the *glmS::gfp* fusion at least as strongly as GlmY, indicating that the cloned fragment of *glmS* mRNA contained the determinants for regulation by either sRNA.

The primary GlmY and GlmZ RNAs are of similar length (184 and 207 nt, respectively), and both sRNAs are subject to 3′ cleavage which yields an approximately 150-nt species [6,9]. Alignment of γ-proteobacterial *glmZ* sequences combined with RNA structure prediction (Figure S1) suggests that GlmZ adopts a secondary structure very similar to that of GlmY RNA, and identified a high degree of sequence identity between the E. coli GlmY and GlmZ RNAs (Figure 1A). Therefore, the two sRNAs seem homologous in sequence and structure, and both promote the accumulation of monocistronic *glmS* mRNA that underlies GlmS overproduction.

### Hierarchy, and Not Redundancy, in the Action of GlmY and GlmZ sRNAs

Given their apparent homology, we tested whether the two sRNAs could complement each other's function. Wild-type E. coli and isogenic Δ*glmY* or Δ*glmZ* mutant strains were transformed with pJV300, pP_L_-GlmY, or pP_L_-GlmZ, and cellular GlmS protein and *glmS* mRNA levels were determined upon growth to early stationary phase (Figure 2). GlmZ, which accumulated as the primary 207-nt RNA, increased *glmS* mRNA and GlmS protein levels in all three genetic backgrounds (lanes 3, 6, and 9). In contrast, GlmY, which accumulated as the processed 148-nt RNA, activated *glmS* only in the wild-type (lane 2) and Δ*glmY* (lane 5) backgrounds, and totally failed to up-regulate *glmS* in the Δ*glmZ* strain (lane 8). This specific failure was also observed when the *glmS::gfp* fusion was used as reporter of *glmS* activation in the tested backgrounds (unpublished data). Plasmid pP_L_-GlmY conferred the same GlmY RNA levels to the Δ*glmZ* as to the other two tested strains (Figure 2), which ruled out the possibility that absence of *glmZ* prevented GlmY accumulation and, consequently, *glmS* activation. Therefore, we concluded that despite their apparent homology, the two sRNAs are not functionally redundant; instead, they act in a hierarchical manner such that GlmY requires GlmZ to bring about *glmS* activation.

Previous work had shown that the proteins Hfq and YhbJ are involved in *glmS* mRNA regulation [4]. We thus expressed GlmY and GlmZ in a larger array of E. coli mutants, including single or combined deletions of *glmY*, *glmZ*, *hfq*, and *yhbJ* (Figure S2). The results, in brief, showed that *hfq* is absolutely required for *glmS* activation by either sRNA, even in the absence of the negatively acting *yhbJ*. Expression of the two sRNAs in Δ*yhbJ* did not further elevate the already high *glmS* mRNA in this background. A Δ*yhbJ* Δ*glmY* Δ*glmZ* triple mutant displayed wild-type *glmS* mRNA levels; in this YhbJ-deficient mutant, GlmZ activated the *glmS* mRNA, whereas GlmY did not. Taken together, these experiments argued against the existence of a *glmZ*-independent pathway of GlmY action on *glmS*. Moreover, they argued that GlmZ acted independently of *yhbJ*, and likely directly upon *glmS* mRNA.

### Mechanism of *glmS* Activation by GlmZ RNA

To understand the obvious hierarchy of the two sRNAs, we next addressed the molecular mechanism by which GlmZ activates the *glmS* mRNA. Work on *rpoS* mRNA activation by DsrA sRNA [16,17] had established an “anti-antisense” mechanism by which an sRNA competes with the formation of a stem-loop structure that normally sequesters the Shine-Dalgarno (SD) sequence of the target mRNA and inhibits its translation. Preliminary RNA structure probing experiments (unpublished data) and in silico prediction by others [4] prompted us to consider an anti-antisense mechanism of *glmS* mRNA activation. That is, the *glmS* SD is predicted to be occluded in a hairpin formed by the 5′ UTR of *glmS* mRNA (Figure 3A, left). We predicted residues 150–157 and 163–169 of GlmZ RNA, which are located in an accessible, single-stranded region (Figure 1A), to base-pair with the 5′ UTR of *glmS* mRNA (Figure 3A, right). This alternative pairing would disrupt the *glmS* hairpin, free the SD, allow ribosome access, and consequently promote *glmS* mRNA translation. Notably, this mechanism would be specific for GlmZ since the residues to interact with *glmS* are not conserved in GlmY species (Figure 1A and [3]).

To test the validity of the above predictions, we mutated the 5' UTR of a *glmS::gfp* fusion. In the resulting mutant, glmS*::gfp, two point mutations (C_−27_ → G and G_−36_ → C; Figure 3A) disrupt the putative GlmZ binding site in the *glmS* 5' UTR while leaving the nucleotides required for the formation of the putative inhibitory hairpin intact. We subsequently measured the effect of these mutations on fusion activation by pP_L_-GlmZ, and observed that the glmS*::gfp fusion was no longer activated (Figure 3B and 3C), suggesting that GlmZ no longer binds to *glmS* mRNA and promotes its translation. In the reciprocal experiment, we constructed mutant plasmid pP_L_-GlmZ* by altering sRNA residues G_+155_ → C and C_+165_ → G (Figure 3A). While pP_L_-GlmZ* no longer up-regulated the parental *glmS::gfp* fusion or the chromosomally encoded *glmS* mRNA, the compensatory mutations facilitated activation of the glmS*::gfp fusion (Figure 3B and 3C). These results demonstrate that an anti-antisense mechanism underlies *glmS* mRNA activation by GlmZ sRNA.

### Full-Length GlmZ Is the Active RNA Species and Enhances *glmS* Translation

**Figure 4:**
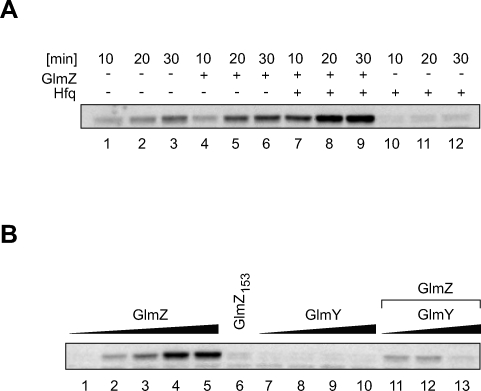
GlmZ, Not GlmY, Enhances *glmS* Translation In Vitro (A) In vitro synthesized *glmS::gfp* fusion mRNA (0.1 μM) was translated with reconstituted 70S ribosomes in the absence (lanes 1–6) or presence (lanes 7–12) of Hfq (0.5 μM) and absence (lanes 1–3 and 10–12) or presence (lanes 4–9) of GlmZ RNA (1 μM). Protein samples were prepared upon incubation for 10, 20, or 30 min, and subjected to western blot analysis using monoclonal α-GFP antibodies to detect the synthesized GlmS::GFP fusion protein. (B) In vitro translation of *glmS::gfp* mRNA (0.1 μM) in the presence of Hfq (0.5 μM) for 15 min. Reactions contained increasing amounts of GlmZ RNA (0, 0.1, 0.2, 0.5, and 1 μM; lanes 1–5), GlmY RNA (0.1, 0.2, 0.5, and 1 μM; lanes 7–10), the 3′ truncated GlmZ_153_ RNA (1 μM; lane 6), or 0.1 μM GlmZ RNA with increasing amouts of GlmY RNA (0.1, 0.2, and 0.5 μM; lanes 11–13). Note that with fixed GlmZ and increasing GlmY concentrations, GlmS synthesis actually decreases (lanes 11–13). This may be explained by competition of GlmY with GlmZ for Hfq-binding, which would inhibit GlmZ-*glmS* mRNA pairing and, consequently, GlmS synthesis.

To obtain direct evidence for translational *glmS* activation, in vitro synthesized *glmS::gfp* fusion mRNA was incubated with reconstituted 70S ribosomes in the presence or absence of GlmZ RNA, and GlmS::GFP fusion protein synthesis was monitored over the course of 30 min (Figure 4A). Since *glmS* activation in vivo strictly requires Hfq (Figure S2 and [4]), the in vitro reactions were also performed in the presence of purified Hfq protein. We found that in the presence of 10-fold excess of GlmZ RNA over target mRNA, GlmS::GFP protein synthesis was activated 1.8-fold (comparing 20-min time points; lane 2 versus 5). However, when Hfq was added to the reaction (lanes 7–9), GlmZ promoted 14.8-fold activation of *glmS::gfp* translation (compare lanes 8 and 11; note that Hfq by itself rather repressed mRNA translation). To the best of our knowledge, this is the first time that translational mRNA activation by a regulatory RNA has been demonstrated in vitro.

### The GlmY and Processed GlmZ RNAs Are Not Activators In Vitro

We had previously established that in vivo, the primary GlmZ transcript is subject to 3′ end RNA processing, which generates a 153-nt species, here named GlmZ_153_ [9]. Kalamorz et al. [4] observed that GlmZ RNA processing is abrogated in a Δ*yhbJ* strain, and concomitantly, full-length GlmZ and *glmS* mRNA accumulate; this led the authors to hypothesize that only full-length GlmZ, and not GlmZ_153_, regulates *glmS* mRNA. Indeed, sequence inspection shows that GlmZ_153_ lacks the above proposed *glmS* target binding site (Figures 1A and 3A). Similarly, GlmY does not possess the *glmS* target site (Figure 1A). Consequently, both GlmZ_153_ and GlmY should be silent in our in vitro system of *glmS* activation. To validate this prediction, *glmS::gfp* mRNA was translated for 15 min along with the full-length GlmZ, GlmZ_153_, or GlmY RNAs (Figure 4B). Quantification of GlmS::GFP confirmed that *glmS::gfp* mRNA was activated by GlmZ in a dose-dependent manner (lanes 1 to 5), and that 10-fold excess of GlmZ over mRNA strongly promoted *glmS::gfp* translation (lane 5). In contrast, 10-fold excess of the GlmZ_153_ (lane 6) or GlmY (lane 10) RNAs failed to promote *glmS::gfp* translation. Moreover, addition of GlmY to reactions spiked with full-length GlmZ RNA did not further elevate protein synthesis (lanes 11–13). Thus, only full-length GlmZ RNA is a direct *glmS* activator, and GlmY most likely achieves *glmS* activation by a translation-independent mechanism.

### GlmY Promotes the Accumulation of Full-Length GlmZ RNA

**Figure 5:**
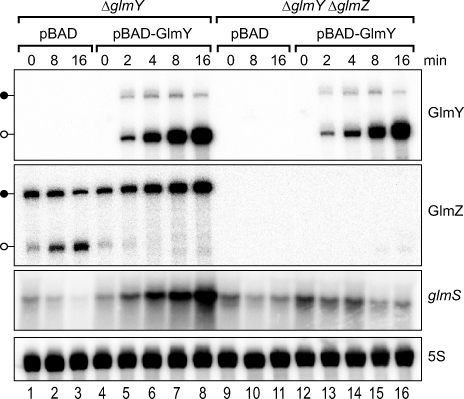
Induction of GlmY Expression Antagonizes GlmZ RNA Processing E. coli MC4100 Δ*glmY* (JVS-8030) or Δ*glmY* Δ*glmZ* (JVS-8113) strains were transformed with control vector, pBAD, or an arabinose-inducible *glmY* expression plasmid, pBAD-GlmY (as indicated above the panels). Transformants were grown to early stationary phase and treated with 0.2% l-arabinose to induce *glmY* expression. RNA was prepared prior to or at the indicated time intervals (in minutes) upon induction, and subjected to northern analysis to detect changes in GlmY, GlmZ, and *glmS* mRNA expression. Black lollipops indicate the primary GlmY or GlmZ transcripts, whereas open lollipops represent the approximately 150-nt processed species of these sRNAs.

Given the above results, we speculated that GlmY activated *glmS* indirectly by means of increasing the amount of the full-length GlmZ RNA. If so, GlmZ levels should be elevated upon GlmY expression, which is indeed observed in bacteria carrying plasmid pP_L_-GlmY (compare lanes 1 and 2 in Figure 2). To determine whether GlmY counteracts GlmZ RNA processing, we studied the effects of induced *glmY* expression. To this end, *glmY* was cloned under control of an arabinose-inducible P_BAD_ promoter, and the resulting plasmid, pBAD-GlmY, or an “empty” pBAD control vector was introduced into E. coli Δ*glmY*. The resulting strains were cultured to early stationary phase, and *glmY* expression was induced with arabinose (Figure 5). Northern blot analysis of samples from the pBAD control strain extracted within 16 min of induction (lanes 1–3) showed that the inducer arabinose itself had a considerable effect on GlmZ and *glmS* expression. That is, it induced GlmZ RNA processing (see position of open lollipop for GlmZ in Figure 5), and reduced the levels of both full-length GlmZ RNA and *glmS* mRNA. (Note that this effect is not observed using an IPTG-inducible system; see Figure S3. Since the *araD* mutation of MC4100 impairs arabinose metabolism [18], this indicates that accumulation of phosphorylated sugar may serve as input signal for the GlmYZ/*glmS* regulatory circuit.) In contrast, induction of GlmY expression from plasmid pBAD-GlmY fully suppressed GlmZ RNA processing (lanes 4–8); moreover, concomitant to the appearance of GlmY RNA, the levels of both the GlmZ full-length species and the *glmS* mRNA increased. Interestingly, induction of pBAD-GlmY (lanes 4–8) primarily yields the processed GlmY species (148 nt), thus, cleavage of the primary GlmY RNA (184 nt) seems to occur rapidly and completely. The same experiment, but performed in a Δ*glmY* Δ*glmZ* double mutant, confirmed that GlmY-dependent *glmS* mRNA accumulation strictly requires the presence of GlmZ (lanes 9–16).

### GlmY Is Essential for GlmZ Stabilization and GlmS Overproduction in a *pcnB* Mutant

**Figure 6:**
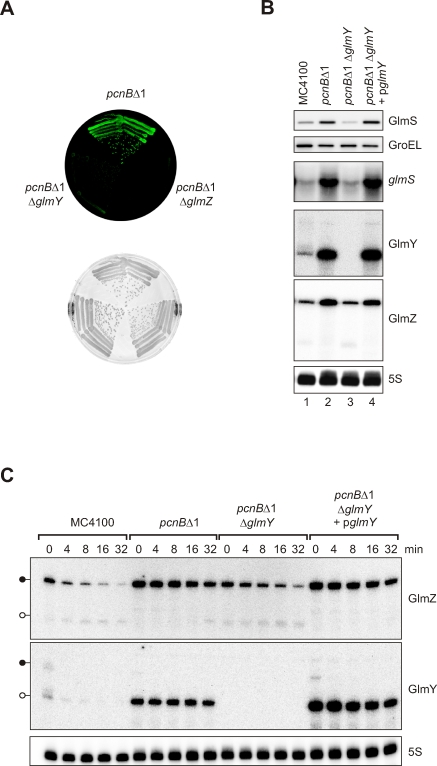
GlmY and GlmZ Accumulate in a *pcnB* Mutant and Promote GlmS Synthesis (A) Activity of the *glmS::gfp* fusion in strains JVS-2058 (*pcnB*Δ1), JVS-8217 (*pcnB*Δ1 Δ*glmY*), or JVS-8242 (*pcnB*Δ1 Δ*glmZ*). Shown are images of an LB agar plate obtained in the fluorescence mode (top) or the visible light mode (bottom). (B) GlmY is essential for GlmS overproduction upon loss of PAP I–mediated polyadenylation. Western and northern blot analysis to detect GlmS protein, *glmS* mRNA, and GlmY and GlmZ sRNAs (as indicated) in the same strains as in (A), but without the *glmS::gfp* fusion plasmid, and additionally, the *pcnB*Δ1 Δ*glmY* double-mutant strain complemented with plasmid p*glmY* (carrying the *glmY* gene with its own promoter). Samples were prepared from early stationary phase cultures. (C) Northern blot analysis of GlmY and GlmZ RNA decay upon transcription block in the same strains as in (B). Bacteria were grown to early stationary phase, and RNA samples were withdrawn prior to or at the indicated time-points (in min) upon treatment with the inhibitor, rifampicin.

Given that GlmY antagonized the processing/degradation of GlmZ, it was likely that GlmZ accumulation resulted from increased GlmZ RNA stability. To prove GlmY-dependent RNA stability changes, we sought to identify a genetic background in which the GlmZ RNA and *glmS* mRNA levels change in response to presence or absence of the chromosomal *glmY* gene. Generally, two mutations were known to alter *glmS* mRNA levels, i.e., Δ*yhbJ* and *pcnB*Δ1 [4,5]. Deletion of *glmY* in these mutant backgrounds (shown for *pncB*Δ1 in Figure 6A) revealed that *glmY* is strictly required for *glmS* mRNA activation in *pcnB*Δ1, as judged by a loss of *glmS::gfp* fusion activity in the *pcnB*Δ1 Δ*glmY* double mutant. Northern blot probing (Figure 6B) confirmed that *glmS* mRNA highly accumulated in *E. coli pcnB*Δ1, and also revealed a strong increase in GlmY and GlmZ RNA levels in the absence of polyadenylation (lane 2). However, the *pcnB* effect is totally lost upon *glmY* deletion (lane 3), suggesting that PAP I acts upstream of GlmY. Ectopic *glmY* expression restored the *pcnB* effect to the *pcnB*Δ1 Δ*glmY* double mutant strain (lane 4).

Rifampicin-treatment experiments showed that the accumulation of processed GlmY and of full-length GlmZ in *pcnB*Δ1 cells was accompanied by increased stability of these RNA species (Figure 6C). Specifically, the half-life of GlmY was increased from approximately 4 min (MC4100) to greater than 30 min (*pcnB*Δ1). Likewise, GlmZ was stabilized approximately 5-fold (∼4 min vs. ∼20 min). In addition, GlmZ RNA processing was suppressed. If GlmY acts as a stabilizer of GlmZ RNA, deletion of *glmY* in the *pcnB*Δ1 strain should reverse the aforementioned GlmZ stabilization. In line with this prediction, GlmZ half-life was reduced to approximately 8 min in the *pcnB*Δ1 Δ*glmY* double mutant. Upon complementation of the *pcnB*Δ1 Δ*glmY* strain with a *glmY* expression plasmid, GlmZ again decayed with the same half-life (∼20 min) as in the *pcnB*Δ1 single mutant. Together with the observation that GlmY expression from plasmid pP_L_-GlmY cannot bypass a Δ*glmZ* mutation in *pcnB*Δ1 (unpublished data), these experiments corroborated that GlmY RNA activates *glmS* mRNA by antagonizing GlmZ RNA decay.

### Loss of GlmY RNA Polyadenylation Causes GlmS Overproduction in *E. coli pcnB*Δ1

GlmS overproduction in *E. coli pcnB*Δ1 had been postulated to result from stabilization of no longer 3′ polyadenylated *glmS* mRNA [5]. However, the fact that a *glmS::gfp* fusion having a different 3′ end was equally activated in *pcnB*Δ1 (Figure 6A) was not in favor of this postulate. Moreover, the Δ*glmY* and Δ*glmZ* mutations abolished *glmS* mRNA accumulation in *pcnB*Δ1 (Figure 6B and unpublished data), yet it was hard to conceive how the GlmYZ sRNAs could affect polyadenylation of *glmS* mRNA.

**Figure 7:**
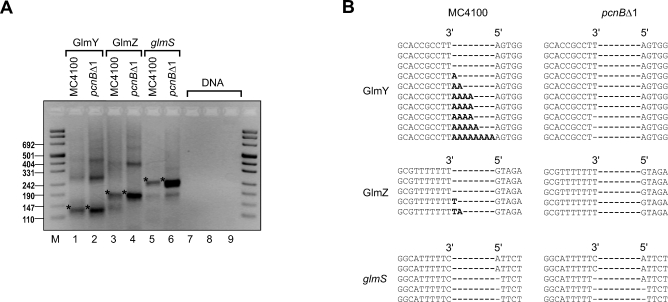
GlmY, and Not GlmZ or *glmS* RNAs, Is Polyadenylated In Vivo (A) Electrophoretic separation (3% agarose gel) of PCR products obtained on cDNA of 5′–3′ circularized RNA. Total RNA was prepared from E. coli MC4100 or its isogenic *pcnB*Δ1 mutant (JVS-2058). PCR products corresponding to the expected sizes of processed GlmY RNA (lanes 1 and 2; 130 bp), full-length GlmZ RNA (lanes 3 and 4; 184 bp), and monocistronic *glmS* mRNA (lanes 5 and 6; 215 bp) are denoted by an asterisk (*). Control reactions were preformed using the same primer pairs as for GlmY (lane 7), GlmZ (lane 8), or *glmS* (lane 9) but with E. coli DNA as a template. Band sizes of a comigrating DNA marker (M) are given to the left. (B) Sequence analysis upon cloning of the above PCR products. Alignment of ten GlmY-specific insert sequences reveals the existence of non-templated adenosine residues (nucleotides in bold-face) in cDNA from wild-type E. coli. The absence of these poly(A) tails in *pcnB*Δ1 cDNA suggests that GlmY undergoes PAP I–mediated polyadenylation. In contrast, no such poly(A) tails were found in GlmZ-specific or *glmS*-specific cDNA insert sequences (five inserts were sequenced for each primer pair and cDNA).

To determine whether GlmY, GlmZ or *glmS* transcripts were polyadenylated, we used an RNA circularization assay previously developed to assess the poly(A) status of chloroplast RNAs [19,20]. Briefly, total RNA of the MC4100 and *pcnB*Δ1 strains was treated with tobacco acid pyrophosphatase (to convert primary 5′ triphosphate to monophosphate groups), and subjected to 3′/5′ end circularization by T4 RNA ligase, and cDNA synthesis. Subsequently, 3′/5′ junction fragments were amplified by PCR (Figure 7A), cloned, and sequenced. This analysis revealed homopolymeric tails composed of up to eight adenosines at the 3′ end of the processed GlmY RNA (148-nt species) in cDNA of MC4100; no such poly(A) tails were found in cDNA from *pcnB*Δ1 cells (Figure 7B). In contrast to GlmY, no poly(A) tails were detected at the 3′ ends of GlmZ or *glmS* RNA, irrespective of whether MC4100 or *pcnB*Δ1 cDNA was analyzed (Figure 7B). Thus, GlmY seems to be the only RNA for which differential stabilities and expression levels in wild type versus *pcnB*Δ1 (Figure 6B and 6C) correlate with the presence or absence of 3′ end poly(A) tails. Therefore, GlmZ stabilization and, consequently, GlmS overproduction in *pcnB*Δ1 are best explained by a loss of polyadenylation of the processed GlmY RNA.

## Discussion

RNA-based regulatory mechanisms are commonly used to control the expression of metabolic genes. In addition to transcriptional and translational attenuators [21], *trans*-encoded sRNAs and *cis*-encoded metabolite-sensing aptamers have increasingly been recognized as mRNA regulators in metabolic pathways. Whereas *cis*-encoded metabolite sensors are prevalent in Gram-positive species, few such elements are known in Gram-negative bacteria [2,22–24]. Conversely, the number of E. coli sRNAs has come close to one hundred [25,26], and fewer sRNAs have been reported from Gram-positive species. Conforming to this obvious bias, the posttranscriptional control of *glmS* relies on a *cis*-encoded RNA sensor in B. subtilis, and on *trans*-encoded riboregulators in E. coli. Interestingly, this is reminiscent of the distinctly different RNA-based mechanisms controlling expression of the tryptophan biosynthetic operon in these two model bacteria [21]. In both cases, i.e., *glmS* and tryptophanase genes, the riboregulatory mechanisms facilitate feedback control of the encoded metabolic pathways [1,4,21]. The network of regulatory factors including the conserved GlmY and GlmZ sRNAs (ref. [3] and Figure S1) that controls the *E. coli glmS* mRNA appears to be well suited for this task, for it provides multiple entry points for the environmental signals that determine *glmS* epistasis.

**Figure 8:**
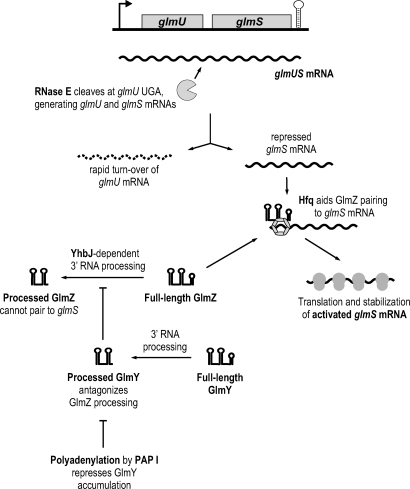
Pathways of *glmS* Activation by GlmY and GlmZ RNAs in E. coli Model summarizing the findings of this and previous studies [3–5]. See Discussion for details.

Figure 8 summarizes the results of this and previous studies [3–5] to suggest a model of how this complex regulatory network may operate in E. coli. NagC-controlled transcription yields a dicistronic *glmUS* mRNA [15] which undergoes RNase E cleavage in the *glmU* stop codon sequence to generate monocistronic *glmS* mRNA [4,5]. This cleavage is independent of the activities of GlmY, GlmZ, Hfq, PAP I, or YhbJ, since the monocistronic *glmS* mRNA is also detected in strains devoid of any of these factors (Figures 1B and S2). Because the remaining *glmU* mRNA lacking an intact stop codon is rapidly degraded, most of the cellular GlmU protein seems to be synthesized from the primary *glmUS* operon mRNA [5]. The *glmS* mRNA is also quickly turned over unless it becomes stabilized by GlmZ. Whether *glmS* mRNA is stabilized due to enhanced translation, the GlmZ/*glmS* RNA interaction, or both, is currently unknown. However, given that the half-life of bacterial mRNA is strongly affected by the association with ribosomes [27,28], increased translation may account for most of the observed *glmS* mRNA stabilization. The RNA chaperone, Hfq, is essential for *glmS* translational activation by GlmZ, and is also known to associate in vivo with both GlmZ [8,29] and *glmS* mRNA (A. Sittka, S. Lucchini, K. Papenfort, C. M. Sharma, J. C. Hinton, and J. Vogel, unpublished data). Regarding *glmS*, the Hfq function seems to be limited to facilitating the GlmZ/*glmS* interaction since an *hfq* mutation does not affect basal *glmS* expression (Figure 1B and [4]) or *glmS* mRNA stability (unpublished data).

GlmZ is the only factor to directly activate *glmS* translation, and we have provided evidence that this process relies upon an anti-antisense mechanism that induces a translation-competent status of the mRNA. Similar to the DsrA-regulated *rpoS* mRNA [16,17], and other sRNA-activated mRNAs [30,31], the anti-antisense mechanism proposed here disrupts an inhibitory hairpin that sequesters the *glmS* RBS. Using purified components, we have been able to reconstitute *glmS* mRNA activation in vitro, proving that the ultimate step in this system requires only the translational machinery and GlmZ RNA.

GlmZ exists in two forms, i.e., the primary 207-nt RNA which can activate *glmS*, and the processed, inactivated GlmZ_153_ species. Transcription of *glmZ* appears to be growth-rate controlled, and to be higher in fast-growing cells [9]. Although the upstream factor(s) that regulate *glmZ* transcription remain unknown, YhbJ acts downstream in the *glmS* activator cascade by regulating the availability of full-length GlmZ at the posttranscriptional level. YhbJ acts as a *glmS* repressor inasmuch as it promotes GlmZ processing [4], and we have demonstrated here that this event generates an inactive GlmZ species. Whether YhbJ, which is conserved in many bacteria [4], is an RNA-binding protein that interacts with GlmZ (or GlmY) is currently unknown, and our attempts to purify soluble YhbJ protein have so far been unsuccessful.

GlmY is also upstream of GlmZ in the herein-described cascade, but in contrast to YhbJ, this RNA acts as a GlmZ/*glmS* activator. In our current working model (Figure 8), GlmY only acts to overcome YhbJ-mediated GlmZ inactivation, which is supported by the observations that GlmY expression has no *glmS* effect in Δ*yhbJ* (Figure S2, lanes 7 and 8) and that *glmS* overexpression is not consistently affected by secondary mutation of *glmY* in the *yhbJ* deletion strain (Figure S4).

At the transcriptional level, the alternative sigma factor, σ^54^, may control *glmY* expression [3]. σ^54^ along with a yet to be identified activator protein could up-regulate *glmY* to bolster up GlmZ action upon nitrogen starvation. Similar to GlmZ, GlmY RNA is also repressed at the posttranscriptional level, albeit by polyadenylation. Loss of this repression in *pncB*Δ1 results in GlmY RNA stabilization and GlmS overproduction. However, under normal growth, the YhbJ-dependent GlmZ processing is likely the rate-limiting step since the GlmZ levels in Δ*yhbJ* are sufficiently high so that GlmY cannot further activate *glmS* expression (Figure S2).

Nonetheless, little is known as to how RNA polyadenylation fluctuates in response to environmental conditions, and certain transcripts have been shown to become differentially polyadenylated in a growth rate–dependent manner [32]. Moreover, greater than 90% of all E. coli mRNAs expressed during exponential growth are controlled by polyadenlyation [33], but the number of mRNAs that are direct PAP I substrates is unknown. Notably, Joanny et al. [5] discussed the possibility that differential expression of regulatory sRNAs may account for some of these PAP I effects, similar to the stabilization of plasmid-borne *cis*-antisense RNAs that underlie alterations of plasmid copy number upon loss of polyadenylation [34,35]. It is worthwhile mentioning that similar to GlmY, additional chromosomal E. coli sRNAs are known to be polyadenylated [9]; at least one of these, SraL, is stabilized in a *pcnB* mutant of *Salmonella* [36]. Thus, it is reasonable to predict that additional mRNAs will be identified as differentially regulated in response to altered sRNA polyadenylation.

The key finding of this paper is that two strikingly similar sRNAs, GlmY and GlmZ, form a regulatory cascade to regulate one and the same target mRNA. Following the original description of the E. coli CsrB and CsrC sRNAs, two homologous antagonists of CsrA protein [37,38], numerous families of homologous sRNAs have been reported (e.g., [8,39]). These include CsrB-like RNAs of other organisms [40], the E. coli OmrA/B sRNAs controlling outer membrane protein biogenesis [41], and a family of four to five *Vibrio* sRNAs regulating quorum sensing [42,43]. The common denominator in these systems is that the sRNAs act redundantly, or at least additively, and can to some degree substitute for each other's function. In contrast, the hierarchy of the sRNAs studied here is novel since GlmY strictly requires GlmZ for *glmS* activation.

Our results have shown that GlmY can antagonize GlmZ 3′ end RNA processing, an event that one may refer to as controlled GlmZ inactivation. Given the related structures of the two sRNAs, this antagonism may involve some kind of molecular mimicry such that GlmY intercepts the factor (YhbJ?) that promotes GlmZ inactivation. If so, this mechanism would bear strong similarity to the activity of small anti-adaptor proteins, e.g., ComS and IraP, which interfere with the controlled proteolysis of transcriptional regulators, ComK or RpoS, respectively [44–46]. In analogy to the studies of ComS or IraP, the development of an in vitro system to study GlmZ degradation will help answer whether GlmY acts by molecular mimicry to save GlmZ from inactivation.

In summary, the GlmYZ system represents a fine example of a regulatory circuit composed of RNA that is no less sophisticated than those composed of proteins. Given the many noncoding RNAs yet to be functionally characterized, we find it reasonable to speculate that hierarchically acting regulatory RNAs will also be discovered in other organisms.

## Materials and Methods

### Media and growth conditions.

Growth in Luria–Bertani (LB) broth (220 rpm, 37 °C) or on LB agar plates at 37 °C was used throughout this study. A culture grown to an optical density at 600 nm (OD_600_) of 2 is referred to as an early stationary phase culture. Antibiotics (where appropriate) were used at the following concentrations: 100 mg/ml ampicillin, 50 mg/ml kanamycin, and 20 mg/ml chloramphenicol. For GlmY expression from pBAD-GlmY, cultures were induced with l-arabinose (0.2% final concentration). Rifampicin (Sigma) was added to the cultures at a final concentration of 500 μg/ml to block transcription.

### Bacterial strains.

The E. coli strains used in this study are listed in Table 1. MC4100 was used as parental wild-type strain. To construct JVS-2058, the *pcnB*Δ1*::kan* allele of SK7988 [47] was moved into MC4100 by P1 transduction. JVS-8018 (Δ*yhbJ::kan*), JVS-8024 (Δ*glmZ::kan*), and JVS-8030 (Δ*glmY::kan*) were constructed by using the one-step gene inactivation protocol with PCR products obtained on pKD4 with primer pairs JVO-1951/-1952, JVO-1947/-1948, and JVO-1661/-1662, respectively (deoxyribonucleotides are listed in Table S1), followed by P1 transduction into MC4100. The *kan* marker of JVS-8018 was eliminated by using the FLP expression plasmid pCP20 [48], and the Δ*glmY::kan* (from JVS-8030), Δ*glmZ::kan* (from JVS-8024), and Δ*hfq::cat* alleles were moved into this strain by P1 transduction to obtain JVS-8076 (Δ*yhb,* Δ*glmY::kan*), JVS-8078 (Δ*yhb,* Δ*glmZ::kan*), and JVS-8146 (Δ*yhbJ,* Δ*hfq::cat*), respectively. Similarly, the *kan* markers of JVS-8076 and JVS-8030 were eliminated using pCP20, and the Δ*glmZ::kan* (from JVS-8024) allele moved into the respective strains to construct JVS-8111 (Δ*yhbJ* Δ*glmY* Δ*glmZ::kan*) and JVS-8113 (Δ*glmY* Δ*glmZ::kan*).

**Table 1:**
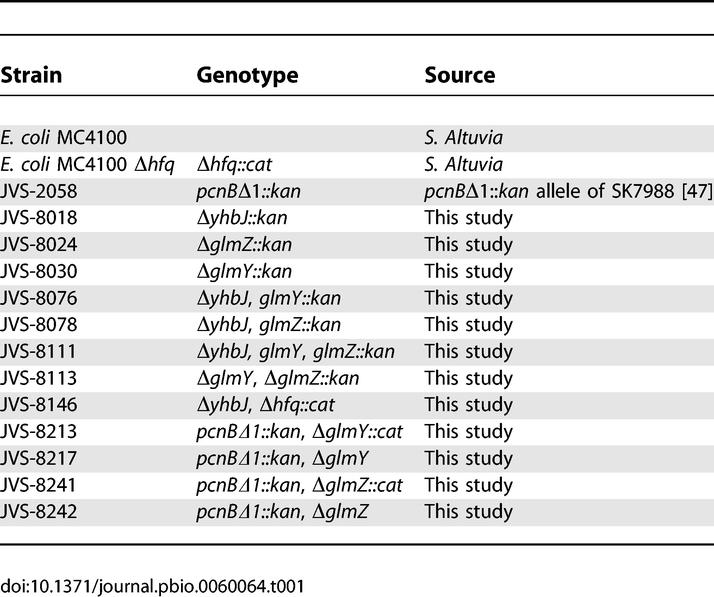
Bacterial Strains

JVS-8213 (*pcnB*Δ1*::kan* Δ*glmY::cat*) and JVS-8241(*pcnB*Δ1*::kan* Δ*glmZ::cat*) were derived from strain JVS-2058 (*pcnB*Δ1*::kan*) by using the one-step gene inactivation protocol with PCR products obtained on pKD3 [48] with primer pairs JVO-1661/-1662 and JVO-1947/-1948, respectively. Elimination of the *cat* markers of JVS-8213 and JVS-8241 resulted in strains JVS-8217 (*pcnB*Δ1*::kan* Δ*glmY*) and JVS-8242 (*pcnB*Δ1*::kan* Δ*glmZ*), respectively.

### Plasmids.

Plasmids used in this study are listed in Table 2. To construct plasmid pP_L_-GlmZ, an *E. coli glmZ* fragment was amplified with oligos jb-103-H and jb-103-I, and inserted by blunt end and EcoRI cloning into vector pZE12-*luc* [49], following a previously published protocol [50]. Plasmid pP_L_-GlmZ* was constructed by introduction of two point mutations into pP_L_-GlmZ by PCR amplification of this plasmid using primers JVO-2609/-2610, followed by self-ligation of the PCR product.

**Table 2:**
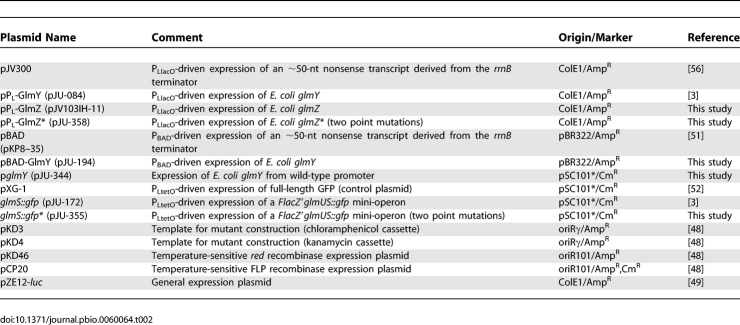
Plasmids

Construction of plasmid pBAD-GlmY (l-arabinose-inducible *glmY* expression) followed the previously published protocol by [51]. Briefly, vector pBAD-His-myc was amplified by PCR using sense primer JVO-0901 (adds an XbaI restriction site upstream of the *rrnB* terminator) and antisense primer JVO-0900 (anneals to the −1 position of the P_BAD_ promoter). A *glmY*-derived PCR product amplified with primers JVO-1399/-0670 was digested with XbaI, and inserted into the above-described plasmid backbone (treated with XbaI) to yield pBAD-GlmY.

For the construction of a low-copy complementation plasmid of *glmY* expression, the *glmY* gene including an approximately 360-nt upstream region was PCR amplified with primers JVO-0670/-2019, the resulting product digested with AatII and XbaI, and ligated to the AatII/XbaI digested plasmid pXG-1 [52] to yield p*glmY*.

The *glmS::gfp* fusion plasmid, pJU-172, which expresses a *FlacZ::glmU-glmS::gfp* mini-operon, was constructed by inserting a BfrBI/NheI-digested PCR product obtained with primers JVO-1270/-1294 into the operon GFP fusion vector pXG-30 [52] digested with the same restriction enzymes. In the resulting plasmid, the C-terminal sequence (17 amino acids [aa]) of *glmU* is fused to the carboxy terminus of a FLAG epitope-tagged, truncated *lacZ* gene (*FlacZ′*). The *glmS* gene (first 7 aa) is fused to the N-terminus of *gfp*. The corresponding glmS*::gfp fusion plasmid, pJU-355, carrying compensatory base-pair changes was constructed by self-ligation of a PCR product obtained on plasmid pJU-172 and using primers JVO-2605/-2606.

### Whole-cell protein fractions and Western blot.

Protein sample preparation and western blotting was carried out as described [52]. An amount of whole-cell protein fractions corresponding to 0.01 OD culture volume was separated by 12% SDS-PAGE and transferred onto a PVDF membrane (PerkinElmer). A polyclonal serum was used 1:5,000 in 3% BSA, TBST_20_ to detect GlmS. GFP and GroEL detection was carried out as described previously [52]. Whenever GlmS and GroEL were detected on the same membrane, blots were first hybridized with α-GlmS, stripped using Roti-Free solution (Roth) according to the manufacturer's protocol, and afterwards hybridized with α-GroEL. Blots were developed using Western Lightning reagent (PerkinElmer), and signals detected with a Fuji LAS-3000 charge-coupled device (CCD) camera.

### RNA isolation and Northern blot detection.

RNA using TRIZOL reagent (Invitrogen) or the Promega SV total RNA isolation kit were performed as described [51,52]. Separation of 5 μg of total RNA (samples prepared with TRIZOL) was on 5% polyacrylamide gels containing 8.3 M urea, or of 12 μg of total RNA (samples prepared with Promega SV totalRNA) on 1.5% agarose gels containing 2.2 M formaldehyde, and northern detection was carried out as described [52]. RNA stability experiments were performed as described in [6]. GlmY, GlmZ, and 5S rRNA were detected using 5′ end-labeled oligodeoxyribonucleotide JVO-1730, −1697, and −0322, respectively. *glmS* mRNA was detected using an RNA probe derived from in vitro transcription of a PCR fragment (primers: JVO-1598/-1599; T7 promoter is added by JVO-1599) in the presence of [α-^32^P] UTP with Ambion's T7 polymerase Maxiscript kit. Signals were visualized on a phosphorimager (Phosphorimager, FLA-3000 Series; Fuji), and band intensities quantified with AIDA software (Raytest).

### In vivo whole-cell colony plate fluorescence imaging.

E. coli strains expressing plasmid-borne *gfp* fusions were streaked on standard LB plates supplemented with the appropriate antibiotics. After overnight growth, colonies were photographed in a FUJI LAS-3000 image analyzer using a CCD camera with a 510-nm emission filter and excitation at 460 nm.

### cDNA synthesis and cloning for 3′ end sequencing.

The procedure for RNA 3′ end sequencing has been described previously [20] and was used with some modifications. A total of 8 μg of DNA-free total RNA prepared from early stationary phase cultures was treated with ten units tobacco acid pyrophosphatase (Epicentre) for 30 min at 37 °C to convert 5′ triphosphate groups of primary transcripts to 5′ monophosphates. Following organic extraction, RNA was treated overnight at 17 °C with 40 units of T4 RNA ligase (New England Biolabs) to circularize transcripts. Following organic extraction and ethanol precipitation, 1.5 μg of self-ligated RNA was converted to cDNA using 100 pmol random hexamer primers and the Superscript III (200 units) reverse transcription kit (Invitrogen) in a 20-μl reaction. Herein, 10-min incubation at 25 °C was carried out before addition of the reverse transcriptase, followed by four subsequent 15-min incubation steps at 42 °C, 50 °C, 55 °C, and 60 °C. After heat inactivation of the reverse transcriptase for 5 min at 85 °C, samples were treated with one unit of RNase H (New England Biolabs) at 37 °C for 20 min. A total of 1 μl of the reaction served as template in a subsequent standard 25-μl PCR reaction using 0.5 units *Taq* polymerase (New England Biolabs) and primer pairs designed to amplify products representing successful self-ligated GlmY (primer JVO-2349/-2355), GlmZ (primer JVO-2350/-2356), and *glmS* (primer JVO-2022/-2388) transcripts. PCR products were separated by 3% agarose gel electrophoresis, and fragments of the expected size were excised, purified, and cloned into the pCR 2.1-TOPO vector (Invitrogen). Colony PCR products obtained on positive clones with standard primers M13 Forward and M13 Reverse were sequenced with primer M13 Reverse.

### Primer extension.

The processing site within the dicistronic *glmUS* mRNA was determined by primer extension according to the SuperscriptIII protocol (Invitrogen). In brief, 10 μg of total RNA prepared with the Promega SV total RNA isolation kit from cultures grown to an OD_600_ of 2 were incubated with 2 pmol of 5′ end-labeled primer JVO-2022 (anneals to positions −19 to −2 relative to the *glmS* start codon) for 5 min at 65 °C, followed by 5 min on ice. The samples were adjusted to a final volume of 20 μl, containing 1× first strand buffer, 0.5 mM each of dATP, dGTP, dCTP, and dTTP, 5 mM DTT, 20 units superasin RNase inhibitor (Ambion), and 200 units SuperscriptIII (Invitrogen). Reverse transcription was carried out for 60 min at 50 °C, followed by inactivation of the enzyme at 70 °C for 15 min. After treatment with 2 units RNaseH (New England Biolabs), 3 μl of the reactions was separated on a denaturing 6% polyacrylamide gel containing 7 M urea. The sequencing ladder was established by using the CycleReader DNA sequencing kit (Fermentas) according to the manufacturer's protocol with 5′ end-labeled primer JVO-2022 on a PCR product obtained on E. coli K12 DNA with primer pair JVO-1177/-2022.

### In vitro translation assay.

Translation reactions were carried out using PURESYSTEM (PGM-PURE2048C; Cosmo Bio Co.) according to the manufacturer's instructions. The 10-μl reactions contained, in addition to 70S ribosomes, 1 pmol *glmS::gfp* mRNA template and Hfq, as well as GlmY and GlmZ sRNA variants (see Figure 4 legend for final concentration). RNA was denatured 1 min at 90 °C and chilled on ice for 5 min before Hfq was added and preincubation at 37 °C was allowed to proceed for 10 min. Puresystem mix was added and incubation continued at 37 °C for the time indicated in the figure legend. Reactions were stopped with four volumes of ice-cold acetone, kept on ice for 15 min, and proteins were collected by centrifugation (10,000 *g*, 10 min, 4 °C). Proteins were detected by western blot analysis with monoclonal antibodies against GFP as described [52].

RNAs were synthesized in vitro using the AmpliScribe T7-*flash* kit (Epicentre) according to the manufacturer's instructions. Templates for *glmS::gfp*, GlmY, GlmZ, and GlmZ_153_ T7 in vitro transcription were established by PCR on plasmids pJU-172 (for T7-*glmS::gfp*), pP_L_-GlmY (for T7-GlmY), and pP_L_-GlmZ (for T7-GlmZ and T7-GlmZ_153_) using primer pairs JVO-1866/-pZE-T1, JVO-1560/-1562, JVO-2130/-2427, and JVO-2130/-2528, respectively.

## Supporting Information

Figure S1 Conservation of Enterobacterial *glmZ* Genes (A) Alignment of *glmZ* genes identified in different enterobacteria. The transcriptional start site (+1) and a putative transcriptional terminator (arrowheads) are denoted for the *E. coli glmZ*. A putative −10 element of σ^70^-type promoters is underlined. Sequences were collected from the following genomes (accession numbers in parentheses): E. coli K12 (NC_000913), Shigella flexneri 2a strain 301 (NC_004337), Salmonella typhimurium LT2 (NC_003197), Yersinia pestis CO92 (NC_003143), Erwinia carotovora subsp. atroseptica (NC_004547), and Photorhabdus luminescens subsp. laumondii (NC_005126), and the unfinished genome sequences of Klebsiella pneumoniae strain Kp342 (http://www.tigr.org) and of Serratia marcescens strain Db11 (http://www.sanger.ac.uk). The alignment was computed with Multalign (http://bioinfo.genopole-toulouse.prd.fr/multalin/multalin.html; [53]).
(B) Secondary structure of E. coli GlmZ RNA predicted by Mfold [54]. Residues that are conserved in the GlmZ RNAs shown in (A) are shadowed. Vertical arrows indicate previously mapped 3′ processing sites [9]. The residues predicted by the RNA-Hybrid program [55] to interact with the *glmS*-leader are highlighted in red.
(54 KB PDF)

Figure S2 Effect of GlmY and GlmZ Expression in Different E. coli Mutant Strains Western and northern blot analyses comparing the effects of GlmY and GlmZ expression in MC4100, Δ*hfq::cat*, JVS-8018 (Δ*yhbJ*), JVS-8146 (Δ*yhbJ* Δ*hfq*), JVS-8113 (Δ*glmY* Δ*glmZ*), or JVS-8111 (Δ*yhbJ* Δ*glmY* Δ*glmZ*). Each of these strains carried plasmids pJV300 (“contr” lanes), pP_L_-GlmY (“GlmY” lanes), or pP_L_-GlmZ (“GlmZ” lanes). Whole-cell protein and total RNA samples were prepared from early stationary phase cultures. Western blots were probed with sera specific for GlmS (top panel) or GroEL (loading control; second panel from top) proteins. For northern blot detection of *glmS* mRNA (third panel from top), 12 μg of total RNA per lane was separated on a 1.5% agarose-formaldehyde gel. To detect GlmY and GlmZ, 5 μg of total RNA was separated on a denaturing 5% PAA gel (fourth and fifth panels from top). The 5S rRNA (bottom panel) probing confirmed equal amounts of RNA in each sample. Black lollipops indicate the primary GlmY or GlmZ transcripts; open lollipops the processed, approximately 150-nt RNA species.
(210 KB PDF)

Figure S3 Induction of GlmY Expression from an IPTG-Inducible Promoter E. coli Top10F′ (*lacI* ^q^; Invitrogen) was transformed with control vector, pJV300, or the IPTG-inducible *glmY* expression plasmid, pP_L_-GlmY (as indicated above the panels). Transformants were grown to early stationary phase, and treated with 0.5 mM IPTG to induce *glmY* expression. RNA was prepared prior to or at the indicated time-intervals (in minutes) upon induction, and subjected to northern analysis to detect changes in GlmY, GlmZ, and *glmS* mRNA expression. Black lollipops indicate the primary GlmY or GlmZ transcripts, open lollipops the processed, approximately 150-nt species of these sRNAs.
(60 KB PDF)

Figure S4 Effect of *glmY* and *glmZ* Deletion or Overexpression in *E. coli yhbJ*
(A) Activation of a translational *glmS::gfp* fusion in a Δ*yhbJ* background requires *glmZ*, but not *glmY*. Colony fluorescence of E. coli MC4100 (wt) and mutant strains JVS-8018 (Δ*yhbJ*), JVS-8076 (Δ*yhbJ* Δ*glmY*), JVS-8078 (Δ*yhbJ* Δ*glmZ*) harboring plasmid *glmS::gfp*, or pXG-1 (*gfp* control plasmid). Images were obtained in the fluorescence (left panel) and visual light (right panel) mode.
(B) Western and northern blot analyses (as in Figure S2) comparing the effects of GlmY and GlmZ expression in MC4100, JVS-8030 (Δ*glmY*), JVS-8024 (Δ*glmZ*), JVS-8018 (Δ*yhbJ*), JVS-8076 (Δ*yhbJ* Δ*glmY*), and JVS-8078 (Δ*yhbJ* Δ*glmZ*).
(169 KB PDF)

Table S1 List of Deoxyribonucleotides Sequences are given in 5′ → 3′ direction; “P∼” denotes a 5′ monophosphate.
(76 KB DOC)

## Abbreviations

5′ UTR: 5′ untranslated region
bp: base pair
nt: nucleotide
SD: Shine-Dalgarno
sRNA: small noncoding RNA
