# Supplementary material for: Two Seemingly Homologous Noncoding RNAs Act Hierarchically to Activate glmS mRNA Translation
Source: PLoS Biol. 2008 Mar 18;6(3):e64. doi: 10.1371/journal.pbio.0060064 (PMC2267818; doi:10.1371/journal.pbio.0060064)
Supplement: Supplementary file 1 [file pbio.0060064.sg001.pdf]

Figure S1

A

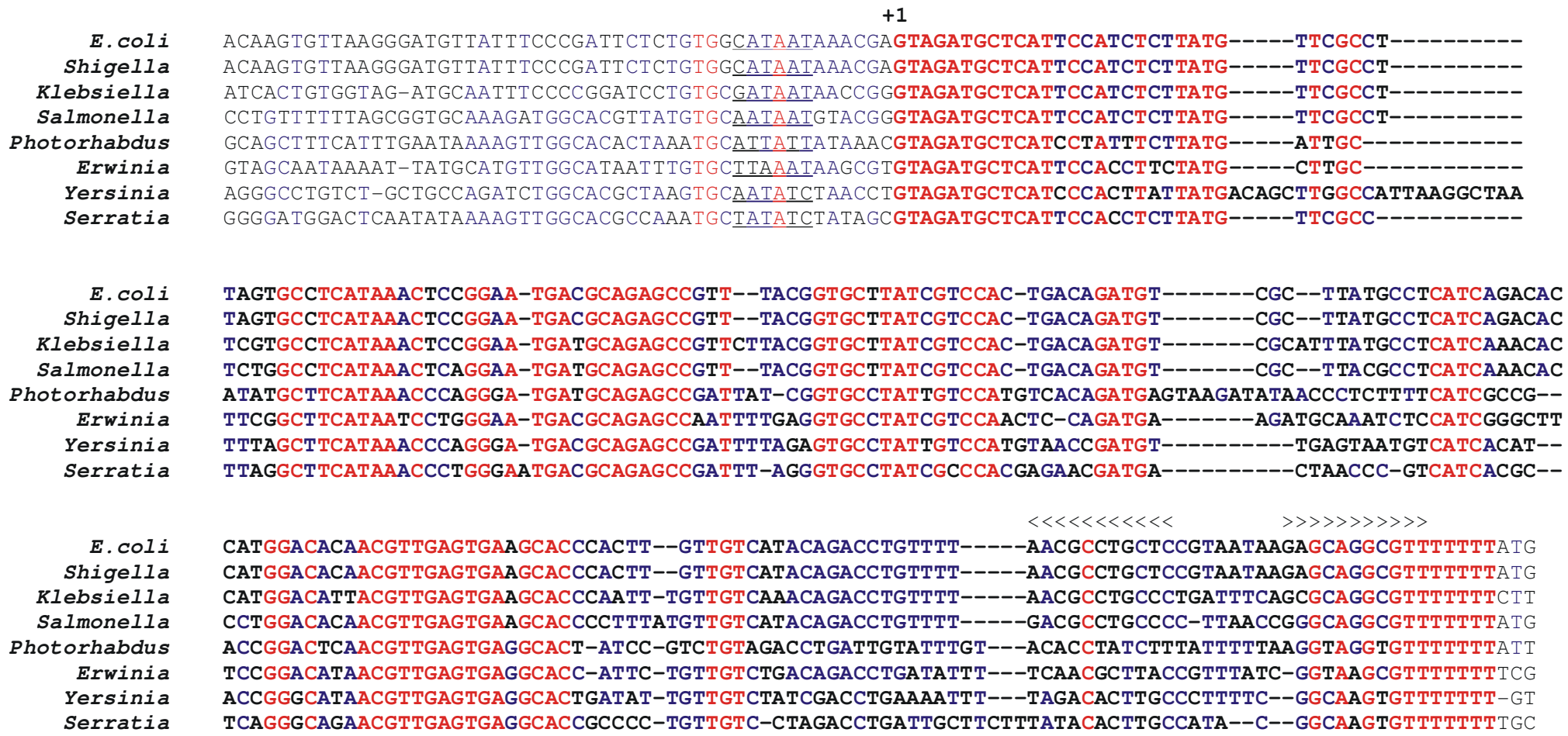

B

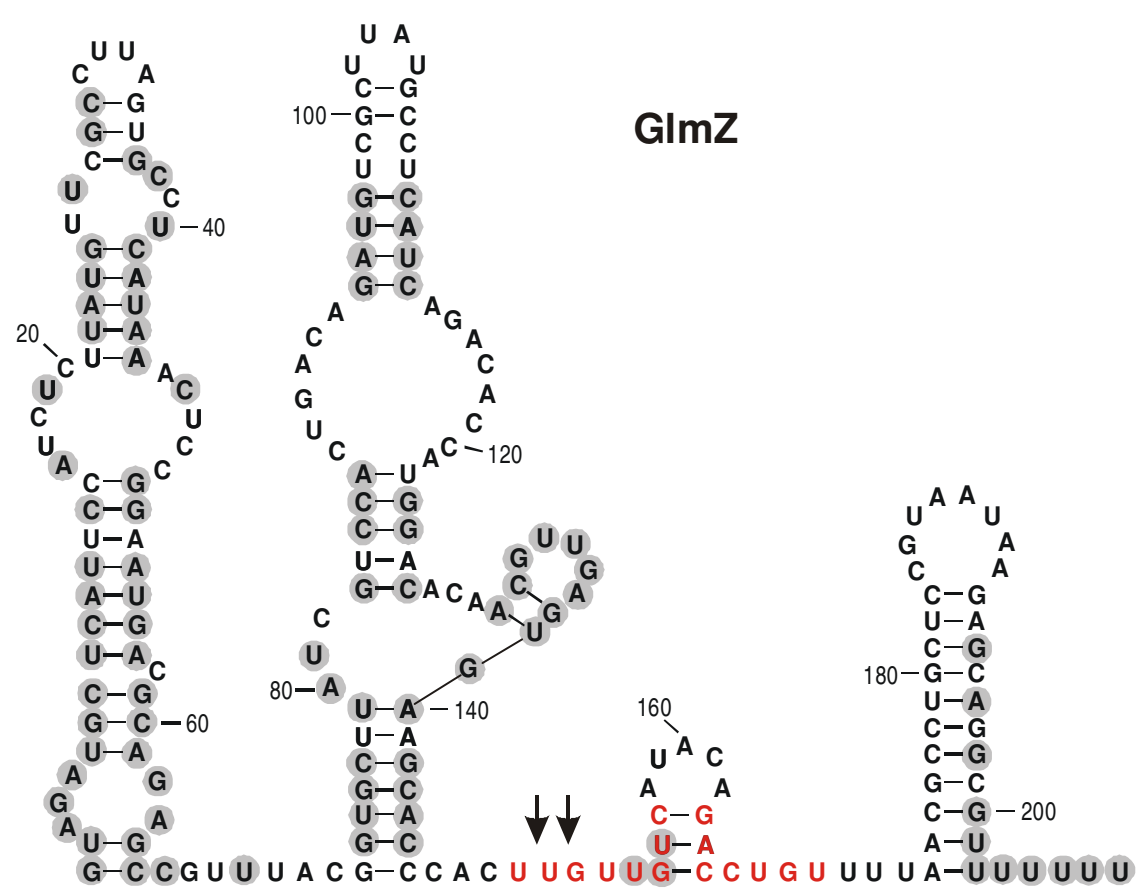

Conservation of enterobacterial *glmZ* genes. (**A**) Alignment of *glmZ* genes identified in different enterobacteria. The transcriptional start site (+1) and a putative transcriptional terminator (arrowheads) are denoted for the *E. coli glmZ*. A putative -10 element of σ^{70}-type promoters is underlined. Sequences were collected from the following genomes (accession numbers in parentheses): *E. coli* K12 (NC_000913), *Shigella flexneri* 2a strain 301 (NC_004337), *Salmonella typhimurium* LT2 (NC_003197), *Yersinia pestis* CO92 (NC_003143), *Erwinia carotovora* subsp. atroseptica (NC_004547) and *Photorhabdus luminescens* subsp. laumondii (NC_005126), and the unfinished genome sequences of *Klebsiella pneumoniae* strain Kp342 (http://www.tigr.org) and of *Serratia marcescens* strain Db11, (http://www.sanger.ac.uk). The alignment was computed with Multalign (http://bioinfo.genopole-toulouse.prd.fr/multalin/multalin.html; [1]). (**B**) Secondary structure of *E. coli* GlmZ RNA predicted by Mfold [2]. Residues that are conserved in the GlmZ RNAs shown in (A) are shadowed. Vertical arrows indicate previously mapped 3´ processing sites [3]. The residues predicted by the RNA-Hybrid program [4] to interact with the *glmS*-leader are highlighted in red.
