# Supplementary material for: Two Seemingly Homologous Noncoding RNAs Act Hierarchically to Activate glmS mRNA Translation
Source: PLoS Biol. 2008 Mar 18;6(3):e64. doi: 10.1371/journal.pbio.0060064 (PMC2267818; doi:10.1371/journal.pbio.0060064)
Supplement: Supplementary file 2 [file pbio.0060064.sg002.pdf]

Figure S2

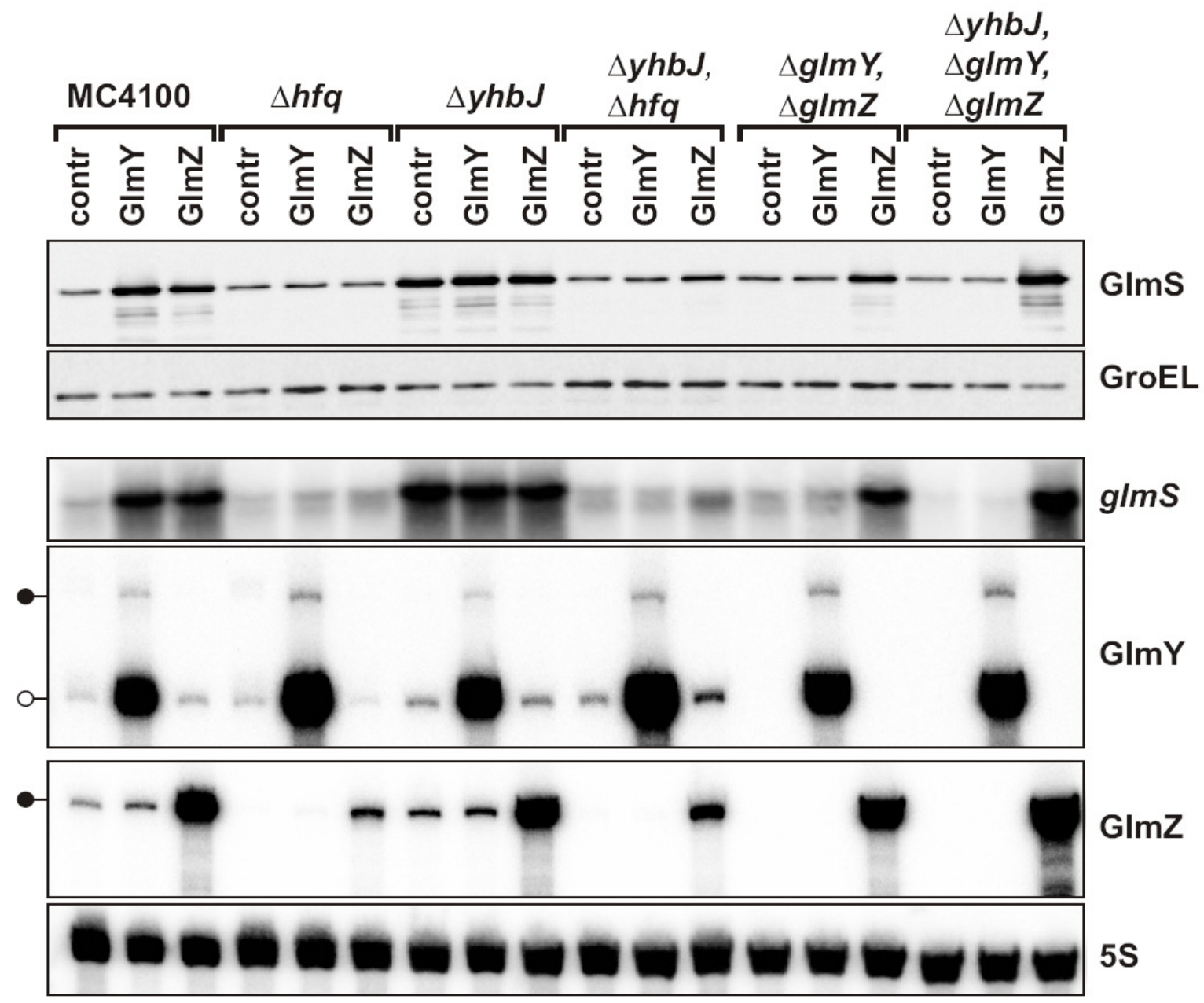

Effect of GlmY and GlmZ expression in different *E. coli* mutant strains. Western and Northern blot analyses comparing the effects of GlmY and GlmZ expression in MC4100, Δ*hfq::cat*, JVS-8018 (Δ*yhbJ*), JVS-8146 (Δ*yhbJ* Δ*hfq*), JVS-8113 (Δ*glmY* Δ*glmZ*), or JVS-8111 (Δ*yhbJ* Δ*glmY* Δ*glmZ*). Each of these strains carried plasmids pJV300 ("contr" lanes), pP_L-GlmY ("GlmY" lanes) or pP_L-GlmZ ("GlmZ" lanes). Whole-cell protein and total RNA samples were prepared from early stationary phase cultures. Western blots were probed with sera specific for GlmS (top panel) or GroEL (loading control; 2nd panel from top) proteins. For Northern blot detection of *glmS* mRNA (3rd panel from top), 12 µg total RNA per lane was separated on a 1.5% agarose-formaldehyde gel. To detect GlmY and GlmZ, 5 µg total RNA was separated on a denaturing 5% PAA gel (4th and 5th panel from top). 5S rRNA (bottom panel) probing confirmed equal amounts of RNA in each sample. Black lollipops indicate the primary GlmY or GlmZ transcripts, whereas open lollipops the processed ~150 nt species of these sRNAs.
