# Supplementary material for: Two Seemingly Homologous Noncoding RNAs Act Hierarchically to Activate glmS mRNA Translation
Source: PLoS Biol. 2008 Mar 18;6(3):e64. doi: 10.1371/journal.pbio.0060064 (PMC2267818; doi:10.1371/journal.pbio.0060064)
Supplement: Supplementary file 3 [file pbio.0060064.sg003.pdf]

Figure S3

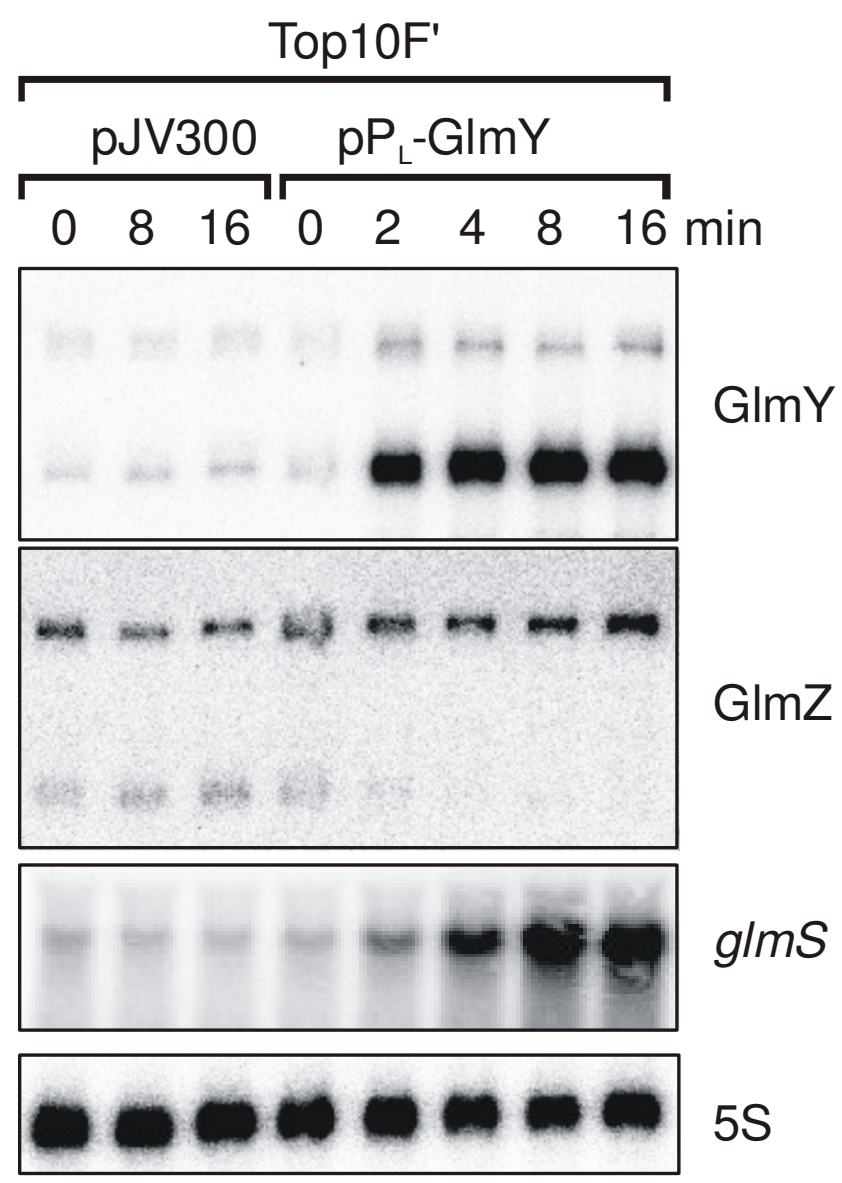

Induction of GlmY expression from an IPTG-inducible promoter. *E. coli* Top10F′ (*lacI*^{q}; Invitrogen) was transformed with control vector, pJV300, or the IPTG-inducible *glmY* expression plasmid, pP$_L$-GlmY (as indicated above the panels). Transformants were grown to early stationary phase, and treated with 0.5 mM IPTG to induce *glmY* expression. RNA was prepared prior to or at the indicated time-intervals (in min) upon induction, and subjected to Northern analysis to detect changes in GlmY, GlmZ and *glmS* mRNA expression. Black lollipops indicate the primary GlmY or GlmZ transcripts, while open lollipops the processed ~150 nt species of these sRNAs.
