# Supplementary material for: Two Seemingly Homologous Noncoding RNAs Act Hierarchically to Activate glmS mRNA Translation
Source: PLoS Biol. 2008 Mar 18;6(3):e64. doi: 10.1371/journal.pbio.0060064 (PMC2267818; doi:10.1371/journal.pbio.0060064)
Supplement: Supplementary file 4 [file pbio.0060064.sg004.pdf]

Figure S4

A

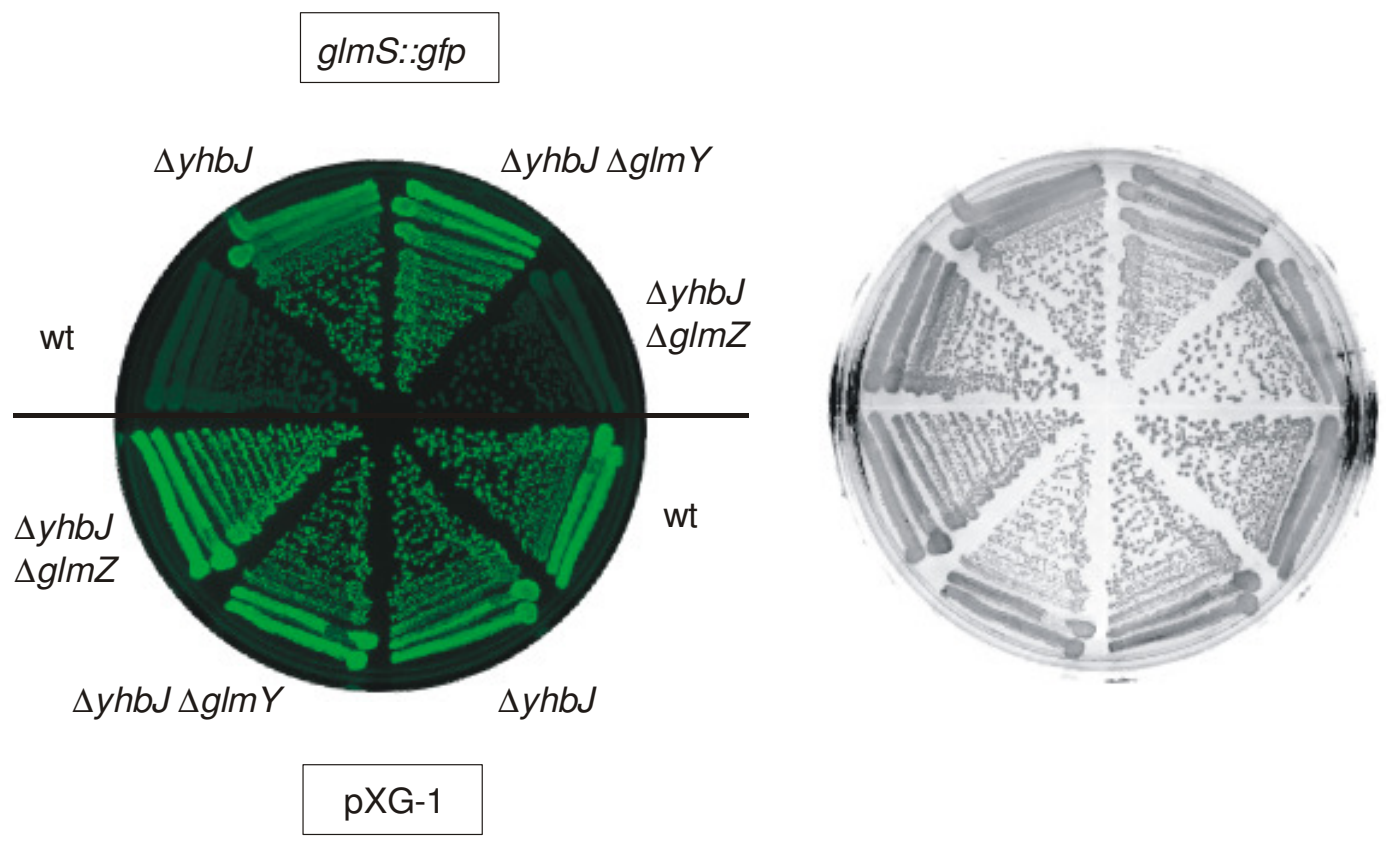

B

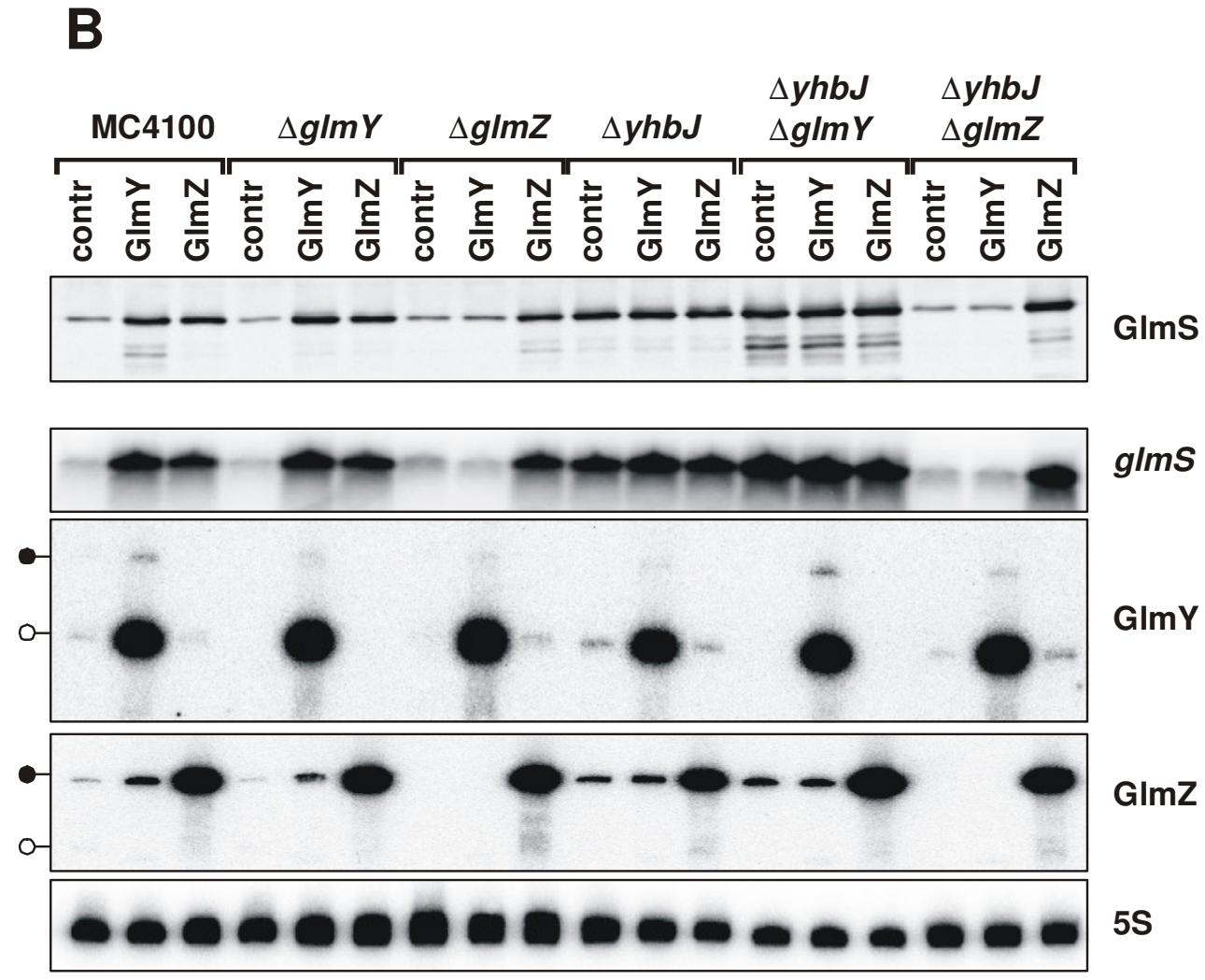

Effect of *glmY* and *glmZ* deletion or overexpression in *E. coli yhbJ* mutant strains. (A) Activation of a translational *glmS::gfp* fusion in a Δ*yhbJ* background requires *glmZ* but not *glmY*. Colony fluorescence of *E. coli* MC4100 (wt) and mutant strains JVS-8018 (Δ*yhbJ*), JVS-8076 (Δ*yhbJ* Δ*glmY*), JVS-8078 (Δ*yhbJ* Δ*glmZ*) harboring plasmid *glmS::gfp* or pXG-1 (*gfp* control plasmid). Images were obtained in the fluorescence (left panel) and visual light (right panel) mode. (**B**) Western and Northern blot analyses (as in Figure S2) comparing the effects of GlmY and GlmZ expression in MC4100, JVS-8030 (Δ*glmY*), JVS-8024 (Δ*glmZ*), JVS-8018 (Δ*yhbJ*), JVS-8076 (Δ*yhbJ* Δ*glmY*), and JVS-8078 (Δ*yhbJ* Δ*glmZ*).
