# Supplementary material for: Two Seemingly Homologous Noncoding RNAs Act Hierarchically to Activate glmS mRNA Translation
Source: PLoS Biol. 2008 Mar 18;6(3):e64. doi: 10.1371/journal.pbio.0060064 (PMC2267818; doi:10.1371/journal.pbio.0060064)
Supplement: Supplementary file 5 [file pbio.0060064.st001.doc]

**Table S1:** List of deoxyribonucleotides (sequences are given in 5' → 3' direction; “P~” denotes a 5’ monophosphate).

| Name | Sequence |
| --- | --- |
| PLlacoB | cgcactgaccgaattcattaa |
| PLlacoD | gtgctcagtatcttgttatccg |
| PZE-T1 | cggcggatttgtcctact |
| jb-103-H | GAAAAAAAGAATTCGGGCCTTCCTGATACATAA |
| jb-103-I | P~GTAGATGCTCATTCCATCTCTTA |
| JVO-0322 | CTACGGCGTTTCACTTCTGAGTTC |
| JVO-0670 | gttttTCTAGACGAATAGCCTGATGCTAACC |
| JVO-1177 | gttttATGCATACAACTGTGACGCGTAATG |
| JVO-1399 | P~AGTGGCTCATTCACCGAC |
| JVO-1560 | gttttttttaatacgactcactataggAgtggctcattcaccgac |
| JVO-1562 | AAGGCGGTGCCTAACTC |
| JVO-1598 | GCGCAACGTGATGTAGC |
| JVO-1599 | gtttttttttTAATACGACTCACTATAGGGAGGAGATGGGCAATCACTTCG |
| JVO-1661 | Cctcaaagcaacgtataatcagtacgttaccaaactattttctttattgggtgtaggctggagctgcttc |
| JVO-1662 | GAGGGGAAGTTCAGATACAACAAAGCCGGGAATTACCCGGCTTTGTTATGggtccatatgaatatcctccttag |
| JVO-1697 | GTGGACGATAAGCACCGTAAACGGCTCTG |
| JVO-1730 | TCAGAAGTTGGACGGCAGG |
| JVO-1866 | gttttttttaatacgactcactataggGATTCTGGCCGGCTAACC |
| JVO-1947 | gtagttccttctcacccggaggcaagcacctccggggccttcctgatacggtccatatgaatatcctccttag |
| JVO-1948 | CTCAGGAAGCAAAGAGGATTACAGAATTATCTCATAACAAGTGTTAAGGgtgtaggctggagctgcttc |
| JVO-1951 | GTAGttattcggtaatgtctcttttagacgttgtgaggagaaacagtacgtgtaggctggagctgcttc |
| JVO-1952 | CGAGCGGAAGTAGTCTGCCAGTTGCTCTGCAATATACACCGAACGGTGCggtccatatgaatatcctccttag |
| JVO-2019 | gttttGACGTCATGGATGCGCATTTTCC |
| JVO-2022 | CGCCAACAATTCCACACATAG |
| JVO-2130 | GTTTTTTTTAATACGACTCACTATAGGGTAGATGCTCATTCCATCTCTTATG |
| JVO-2349 | CTACGCTGATATCAGAAGTTGG |
| JVO-2350 | GACGATAAGCACCGTAAACG |
| JVO-2355 | CGAAACGTCGAGTTAGGCAC |
| JVO-2356 | CTCATCAGACACCATGGACAC |
| JVO-2388 | GCGTAACCTGGCAAAATCG |
| JVO-2427 | AAAAAAACGCCTGCTCTTATTACGGAG |
| JVO-2528 | ACAAGTGGGTGCTTCACTC |
| JVO-2605 | CGAGAACGATATAAATCGGAATCAAAAACTATG |
| JVO-2606 | P~GTCAAGCTGTATGCCCGTTTTTTCG |
| JVO-2609 | P~ATGAGAACAAGTGGGTGCTTCACTC |
| JVO-2610 | ACAGAGCTGTTTTAACGCCTGCTCC |
